# Survey of physicochemical characteristics and microbial contamination in selected food locally vended in Morogoro Municipality, Tanzania

**DOI:** 10.1186/s13104-015-1716-5

**Published:** 2015-11-26

**Authors:** Hezron Emmanuel Nonga, Helena Aminiel Ngowi, Robinson Hammerthon Mdegela, Eliud Mutakyawa, Gabriel Busungu Nyahinga, Robert William, Mtumwa Mohd Mwadini

**Affiliations:** Department of Veterinary Medicine and Public Health, Faculty of Veterinary Medicine, Sokoine University of Agriculture, P. O. Box 3021, Chuo Kikuu, Morogoro, Tanzania; Faculty of Science, Technology and Environmental Studies, The Open University of Tanzania, P.O. Box 31608, Dar es Salaam, Tanzania

**Keywords:** Raw milk, Fruit juice, Raw fish, Bacteria, Morogoro

## Abstract

**Background:**

Raw milk, raw fruit juice and raw fish are enriched with essential nutrients for human diet but are prone to microbial contamination along the value chain. This cross sectional study was conducted to assess physicochemical characteristics and microbial quality of raw milk, fruit juice and fish from food vendors in Morogoro Municipality, Tanzania. The physicochemical assessment of food samples was done by smell, colour, presence of debris, turbidity, consistence, pH and clot on alcohol test. Hygiene of food containers, personnel and the vending environment was also assessed. Qualitative and quantitative microbial assessment of food was done using standard laboratory protocols as described by Tanzania Bureau of Standards and International Systems of Standards.

**Results:**

Raw milk sold in Morogoro was of poor quality since was adulterated with water, contained sediments and clotted on alcohol test. Up to 63 % of the milk samples were contaminated with *Escherichia coli* and 60 % had higher total viable count (TVC) than the recommended values. Raw fruit juice was stored in dirty containers and sold under unhygienic environment. Seventy-three percent of juice samples had TVC beyond the recommendations while *E. coli* contamination rate was 63.3 %. The raw fish samples had started spoiling as depicted through sensory evaluation. *E. coli* contamination rate was 55 % and that of *Campylobacter jejuni* was 0.5 %. The mean TVC of raw fish was 8.1 (Log cfu/g) and 96.2 % of the fish samples had TVC beyond the recommended limits of 5.0 Log cfu/g.

**Conclusions:**

The physicochemical characteristics of food vended in Morogoro Municipality were of poor quality. The food had high bacterial contaminations. This situation poses health risks to the public and losses to food vendors due to spoilage. Stakeholders in food value chain should be educated on safe production and good hygienic practices. Routine quality and safety assessment of locally vended food, inspection of selling premises and regular health check-up of the personnel involved in food vending industry should be instituted.

## Introduction

### Background

Food- and water-borne pathogens are leading causes of illnesses in developing countries, causing deaths to about 1.9 million people annually at the global level [1]. In developed countries, an estimated one-third of the population is affected by microbiological food-borne diseases each year [2]. In Africa, food- and water-borne diseases are responsible for 33–90 % cases of mortality in children [3]. Most of the cases are due to microbial contamination in food which is associated with poor hygiene in handling along food chain especially the street food. However, street food industry plays an important role in developing countries in meeting the food demands of the urban dwellers. Street foods feed millions of people daily with a wide variety of foods that are relatively cheap and easily accessible [4]. According to FAO, street foods have significant nutritional implications for consumers, particularly from middle and low-income sectors of the population [5]. Street food vendors in developing countries are often unlicensed, untrained in food hygiene and sanitation, and work under crude unsanitary conditions [5–8]. In Tanzania, urbanization accompanied with low wages offered to employees and labourers has led to proliferation of street food vendors who offer cheap meals that is always contaminated by microbes [9–11]. In a study to evaluate microbiological quality of ready-to-eat foods in Pemba Island, coliform bacteria, *Salmonella* spp. and *Vibrio alginolyticus* were the common contaminants [9].

Milk as a nutritious food is prone to microbial contamination and many milk-borne epidemics of human are spread through contaminated milk. The primary sources of microbial contamination in milk are the infected or sick lactating animals [12, 13]. The secondary causes of microbial contamination occur along the milk value chain from milk handlers, unsanitary utensils and/or milking equipments and water supplies used in sanitary activities [12, 13]. Studies show that up to 90 % of all dairy—related diseases are due to pathogenic bacteria like *Brucellaabortus*, *Escherichia coli* 0157:H7, *Mycobacterium bovis*, *Campylobacterjejuni* and *Staphylococcusaureus* [14–16]. Moreover, fish is one of the most important protein sources for the rapid growing population in Tanzania. The per capita fish consumption has been estimated to be 25–30 kg [17]. A large population in Tanzania lives nearby water bodies and the consumption of fish constitutes more than 50 % of the total animal protein source [18]. However, fish are prone to microbial contamination from the sources when the water bodies are contaminated and along the value chain [19, 20]. When the fish are contaminated, the microbes accelerate spoilage and are the cause of food-borne diseases to consumers.

In addition, unpasteurized fruit juices pose a high public health risk especially in developing countries where strict food hygiene is limited [21–23]. Studies have reported pathogens like *Salmonella* spp., *Shigella* spp., *E. coli* O157:H7 and *Listeria monocytogenes* as common contaminants in juices with low pH [24, 25]. Most of the locally vended raw fruit juices in Tanzania are characterized with low pH [8, 11], a condition which favour survival of bacteria. The number of documented outbreaks of human infections associated with the consumption of raw fruits, vegetables, and unpasteurized fruit juices has increased in recent years [26, 27]. Unhygienic preparation, processing and handling of the raw fruit juices are the key sources of bacterial contaminations [8, 22]. A study by Simforian et al. [11] reported high microbial contamination in raw fruit juices locally vendered in Dar es Salaam city, something that poses a threat of food-borne diseases to consumers.

There has been a big number of food-borne diseases in Tanzania including diarrhoea, dysentery, typhoid and cholera [28], but it is not known how much of these diseases have been contributed by the consumption of contaminated street vended food like milk, fish and raw fruit juices. Milk, fish and fruits are nutritious food which nutritionists recommend them in the daily diets of humans but when are not well handled; they are the potential sources of food-borne diseases. Over 90 % of the people in Tanzania consume raw milk that predisposes them to infections [29]. In cities like Morogoro, there has been an increase in consumption of freshly extracted fruit juices because of availability of variety of fruits throughout the year [30] but their safety is not known. This study aimed to assess the physical characteristics and establish the bacterial status of the selected food that is locally sold in Morogoro Municipality, Tanzania. The baseline data of this work will provide added information on status of microbial contaminations in food marketed in Tanzania. This will also be useful to public health officials in instituting control measures of food-borne diseases in Morogoro Municipality and Tanzania at large.

## Methods

### Study area

The study was conducted in Morogoro Municipality, Tanzania. Selection of the municipality, as the study area was based on convenience of accessibility from the laboratory and it generally represents food vending systems in urban areas of Tanzania.

### Ethical approval

The permission to carry out this study was granted by the Morogoro Municipal Director. The Vice Chancellor of Sokoine University of Agriculture (SUA) issued a research permit letter on behalf of The Tanzania Commission for Science and Technology (COSTECH) that permitted the researchers from SUA to conduct their research in Morogoro Municipality. Before commencement with the research, the study protocol was prepared, submitted to, and approved by the SUA ethical committee. Verbal consent was obtained from each of the food vendor after explaining the purpose and importance of the study prior to start of data collection. Participation in the study was on voluntary basis. In addition, before the manuscript was submitted to journal for publication, the data were submitted to the local ethics committee at SUA and to the Morogoro Municipal Director and permission to publish the results was granted.

### Sampling and sample handling

Milk samples were collected from farmers, vendors and restaurants/milk kiosks in different wards and streets. At farm level, 20 raw milk samples were collected directly from the storage containers with the pooled milk. From vendors, nine raw milk samples were collected directly from the storage containers. Eleven boiled milk samples were collected from 11 randomly selected restaurants/milk kiosks. Before sampling, milk assessment for smell, colour, any dirty, turbidity, cleanliness of containers and milk handlers was done. The smell of milk was assessed after the farmer or vendor had opened the lid of the container. The colour was examined visually by putting well stirred milk in the clean glass container as described by Kurwijila et al. [29]. About 250 ml of milk sample was collected from each source, put in a sterile glass bottles and placed in a cool box with ice packs during the field work. A total of 40 milk samples were collected and analysed.

The raw juice samples were collected from vendors in different places including 11 samples from the bus terminals, 10 samples from selected cafeterias and 9 samples along roadsides. Before sampling, juice assessment for smell, colour, dirtiness, cleanliness of containers and assessment of the environment was done. The juice samples were collected directly from the storage containers into sterile falcon tubes and placed in a cool box with ice packs before shipment to the laboratory for analysis. A total of 30 juice samples were collected and analysed.

1. Cleanliness of the fruit juice containers:
  Clean: A container is obviously free from dirty substances such as abnormal stains, dried juice spillover on surfaces and has not changed its original colour due to wear and tear.
  Satisfactory: no dried juice spillover, presence abnormal stains that cannot be removed by cleaning, container appears to have irreversibly changed its original colour but appears clean.
  Dirty: refers to presence of dried juice spillover, change of original container colour and presence of abnormal stains that may be removable by cleaning.
2. Cleanliness of the environment at fruit juice selling points:
  Dirty: the area around where juice is sold has discarded litter materials such as plastic bags, papers, poured water on the ground, sold nearby municipal drainage systems, near dump sites or any other materials that should not be discarded irregularly such as maize cobs, mango and orange peelings.
  Clean: the area is free from above mentioned factors dirty environment.
3. Presence of dirty in fruit juice:
  Yes: refers to presence of abnormal suspended particles such as sand, fruit peelings or any other materials that should not be present
  No: refers to lack of obvious abnormal suspended materials.

Note that the following criteria were developed and used in assessment of hygienic practices in the juice handling environment as per FAO [31].

Freshwater and marine fish samples were collected from 14 fish shops and Mindu dam shoreline selling point. Before collection of samples, information on the type of fish (freshwater or marine), fish sources and means of package were collected. From the fish shop freezers, four representative fish samples were taken from different corners, each was put into separate sterile zipped plastic bags and placed in a cool box with ice packs. A total of 185 fish samples were collected from different fish selling points in Morogoro of which, 79 (42.7 %) were marine and 106 (57.3 %) freshwater fish. All the collected samples were being transported in a cool box with ice packs to the Faculty of Veterinary Medicine laboratory at Sokoine University of Agriculture for analysis within 2–3 h of collection.

### Laboratory analysis of food samples

#### Milk samples

##### Physicochemical quality assessment of milk samples

Part of the milk samples was filtered using a clean white cloth and assessed for physical dirt/contaminants [32]. In case of any debris remained on the white cloth; the milk was regarded as dirty. Determination of pH of milk was done by using Jenway 3540 pH and Conductivity Meter (Bibby Scientific Ltd, Staffordshire, UK) which was first calibrated using standard buffer solutions of pH 7.0 and pH 4.0 (ISO 7218:2007) [33]. The used reference pH was 6.6–6.8 for raw cow milk as given by East African Community standards [34]. Alcohol test was done to ascertain the milk acidity and it involved mixing a 5 ml aliquot of the milk sample with an equal volume of 70 % ethanol in a clean test tube and examined for the presence clots. Specific gravity was measured by use of a lactometer at standardized temperature of 20 °C [29]. The used reference relative density for whole milk was 1.027–1.030 as given by East African Community standards [34].

##### Assessment of *Escherichia coli* and total viable count (TVC) in milk samples

Total viable count (TVC) for enumeration of microorganism in raw milk at 37 °C applied a protocol described by Tanzania Bureau of standards and ISO/FDIS 8261 (E) [35, 36]. Briefly, tenfold serial dilution of milk sample from 10^−1^ to 10^−10^ in sterile normal saline solution was done, using disposable pipettes. From each dilution, 1 ml of diluted sample was placed in a sterile Petri dish followed by the addition of 20 ml of molten nutrient agar (Oxoid Ltd, Basingstoke, UK), gently shaken and left to solidify. Incubation was done under aerobic condition at 37 ± 1 °C for 24 ± 3 h. Microbial colon count on the plates was done with the aid of portable magnifying lens and colonies in the culture plate were countered by using colony counter. Two consecutive plates with 30–300 colony forming units (cfu/ml) were considered for record [37]. The countable microbial colonies from two consecutive plates of each sample were converted into colony forming units per millilitre (cfu/ml) using a formula N = ∑C/v × 1.1 × d, where N—number of microbial colonies counted, C—sum of colonies counted in two successful dilutions, v—volume of sample and d—dilution in the first plate counted [33].

*Escherichia coli* was determined in milk samples to assess for the possibilities of faecal contaminations. Briefly, 1 ml of milk sample was added into universal bottle containing 9 ml of buffered brilliant green bile glucose broth (EE broth) (Oxoid Ltd, Basingstoke, UK) then incubated at 37 °C for 24 ± 2 h under aerobic condition for enrichment. Thereafter, the sample was subcultured into tryptone water and incubated under the same conditions. Presence of *E. coli* was confirmed by addition of indole reagent in tryptone water and observed for presence of a red ring in the alcoholic phase which indicated indole production signifying a positive test. Isolation and identification of pure colonies of *E. coli* was done as described in ISO 7251:2005 protocol [38]. Briefly, the enriched sample in EE broth was striped onto MacConkey agar and incubated at 37 °C for 24 ± 2 h under aerobic condition. Typical colonies of *E. coli* grown on MacConkey agar were dry, medium in size, pink in colour and appeared singular or in groups. Gram staining and biochemical reactions like oxidase, nitrate and indole tests were performed to confirm the presence of *E. coli*.

#### Juice samples

##### pH and microbiological determination in juice samples

The pH of juice samples was measured by using Jenway 3540 pH and Conductivity Meter (Bibby Scientific Ltd, Staffordshire, UK) after calibration using standard buffer solutions of pH 7.0 and pH 4.0 [33]. Microbiological evaluation was done by establishing TVC and most probable number (MPN). Total viable count for enumeration of microorganism in raw juice at 37 and 45 °C applied a protocol described by Tanzania Bureau of standards and ISO/FDIS 8261 (E) [35, 36] as was described for the raw milk. For the determination of total coliform count (TCC) (coliform and faecal coliform bacteria), MPN, the presumptive test for coliforms was carried out using MacConkey broth (Oxoid Ltd, Basingstoke, UK) in the three test tube method as described by WHO [39]. The three tubes with 10 ml of broth were inoculated with 1 ml from 10^−1^, 10^−2^, 10^−3^, 10^−4^ and 10^−5^ prepared dilutions and incubated at 37 °C for 24 h for coliforms and at 45 °C for faecal coliforms as described by WHO [39]. All tubes showing gas production were observed and recorded. The MPN tables for three tubes dilution were used to report the result of the presumptive MPN of coliform bacteria per ml. *E. coli* was confirmed in samples as described above for the milk samples.

#### Fish samples

##### Physical assessment of fish samples

Upon arrival in the laboratory, fish samples were identified to species level using standard identification keys. Biodata details of fish like sex, weight, age and length was not determined. Fish samples from the freezers were allowed to thaw followed by physical assessment. Physical evaluations undertaken included smell, sight (appearance) and touch (consistence) as described by Codex guidelines for the sensory evaluation of fish and shellfish in laboratories [40]. A special attention was directed to the eyes, gills, skin, texture and attachment of scales for the scaled fish like tilapia.

##### Microbiological assessment of fish samples

For the purpose of qualitative analysis, three bacteria groups were targeted namely *E. coli*, thermophilic *Campylobacter* and *Salmonella* spp. Isolation and identification of *E. coli* from fish swab samples started with enrichment in buffered brilliant green bile glucose broth (EE broth) (Oxoid Ltd, Basingstoke, UK) and all the other procedures were done as for the milk samples.

Fish swabs with samples for *Campylobacter* isolation were immediately placed in a sterile universal bottle containing 10 ml of Bolton broth (Oxoid Ltd, Basingstoke, UK) with antibiotic (cefoperazone 32 mg/l) supplements (Oxoid Ltd, Basingstoke, UK). The samples in the Preston broth were loaded in the microaerophilic candle jars (Coldstream Engineering Ltd, Arista, Sweden) with a lighting candle and incubated at 37 °C for 24 h for enrichment as described by Atabay and Corry [41]. Isolation and identification of thermophilic *Campylobacter* was carried out according to the method described by Karmali et al. [42]. Briefly, after incubation, the universal bottles with enriched samples were properly shaken and sub-cultured onto modified charcoal cefoperazone deoxycholate agar (Oxoid Ltd, Basingstoke, UK) for primary isolation of *Campylobacter*. The inoculated Petri dishes were incubated at 42 °C for 48 h under microaerophilic conditions and colonies resembling *Campylobacter* were sub-cultured onto blood agar (Oxoid Ltd, Basingstoke, UK) at 37 °C under microaerophilic conditions for 24 h. Suspected *Campylobacter* colonies on blood agar that were Gram negative, curved, or spiral rods and showed corkscrew-like motion; positive to catalase, oxidase and nitrate reduction tests were further tested for hippurate hydrolysis, H_2_S production, and susceptibility to nalidixic acid and cephalothin. These parameters formed the basis for the identification of *C. jejuni*, *C. coli*, or *C. lari* as described by On [43].

The swab samples for *Salmonella* isolation were put in sterile universal bottles containing 10 ml selenite cysteine broth (Oxoid Ltd, Basingstoke, UK). Isolation and identification of *Salmonella* species was carried out by using ISO 6579:2002 protocol [44].

Total viable count (TVC) of microorganisms in fish at 37 °C was performed using a protocol described previously [35]. Briefly, 1 g of fish sample was grinded using sterile mortar and pestles and 10 ml of maximum recovery diluents (MRD) (Oxoid Ltd, Basingstoke, UK) was added and thoroughly mixed as described in EAS 217-1-3:2008 protocol [45]. The TVC in fish sample at 37 °C applied a protocol described by Tanzania Bureau of standards and ISO/FDIS 8261 (E) [35, 36] as was described for the raw milk. After incubation all colonies were counted and the results reported as cfu/g.

### Statistical analysis

The TVC values were log transformed before statistical analysis in order to make the frequency distribution more symmetrical. The Epi info version 2000 (approved by CDC, Atlanta, GA, US) was used for statistical analysis. Differences on means of TVC between groups of samples were compared by Mann–Whitney/Wilcoxon two-sample test. Means were considered significantly different at *p* < 0.05.

## Results

### Physicochemical and microbiological quality of milk samples

Total of 40 milk samples were collected, whereby 11 were boiled milk and 29 were raw milk from Morogoro Municipality. It was found that the milk sold in Morogoro Municipality was of poor quality as depicted through physical and microbiological assessment. Three milk samples (7.5 %) had bad smell, 20 % were contaminated with cow dung, hairs and unidentified sediments, 25 % clotted on alcohol test and were the milk which had pH between 5.7 and 6.1. Majority (70 %) of the milk samples had low specific gravity (Table 1).

**Table 1:** Physicochemical quality assessment of milk samples

Variables assessed	Category	Frequency	Percentage
Colour	Milky creamy white	40	100.0
	Abnormal	0	0.0
Smell	Normal milk smell	37	92.5
	Bad smell	3	7.5
Cleanliness	Clean milk, no dirty	32	80.0
	Dirty present (dung, hairs and unidentified segments)	8	20.0
Clot on addition of 70 % ethanol	Not clotted	30	75.0
	Clotted	10	25.0
Specific gravity	Normal (1.027–1.030)	12	30.0
	Below normal	28	70.0
pH, (normal is 6.5–6.7)	5.7	8	20.0
	6.1	3	7.5
	6.6	15	37.5
	6.7	10	25.0
	6.8	4	10.0

Microbiological results showed that the mean TVC of raw milk was 4.7 (Log cfu/ml) with the highest count recorded in raw unboiled milk samples (5.5–5.6 Log cfu/ml) particularly those which had bad smell and sediments (Table 2). Lowest microbial count (3.3–4.1 Log cfu/ml) was depicted in five boiled milk samples and three samples which had a pH of 5.7. Assessment of different sources of milk samples against differences in the means TVC showed the significant differences (*p* = 0.0000) with the highest mean TVC (4.9 Log cfu/ml) recorded in milk from vendors. The mean TVC (4.9 Log cfu/ml) in unboiled milk was higher than that of boiled milk (4.3 Log cfu/ml) and the difference was statistically significant (*p* = 0.0000). The figures described herewith are averages over all target categories of milk sources in this study as were depicted in Table 2. The overall results indicated that 60 % of all milk handled had higher TVC than the maximum recommended of 5.3 Log cfu/ml for raw milk as given by East Africa Community standards [34]. A total of 25 (62.5 %) of the milk samples were contaminated with *E. coli*. This included all the eight milk samples from the farms which had sediments of dung and hairs.

**Table 2:** Sources and nature of milk against TVC and *E. coli* contaminations (n = 40)

Factor assessed	Category	Mean TVC (Log cfu/ml)	p value	Number of milk contaminated by *E. coli*	Percentage	p value
Source of milk sample	Farm (n = 20)	4.8	0.0000	14	35.0	0.3848
	Vendors (n = 9)	4.9		6	15.0	
	Restaurant/Kiosks (n = 11)	4.2		5	12.5	
Nature of milk	Raw unboiled (n = 29)	4.9	0.0000	20	50.0	0.3146
	Boiled (n = 11)	4.3		5	12.5	

Note that the maximum recommended TVC in raw milk according to East Africa Community standards [34] is 5.3 Log cfu/ml. The percentages of milk contaminations were calculated out of 40 samples analysed

### Physicochemical and microbiological quality of juice samples

A total of 30 samples of locally vendered raw fruit juices from: cafeteria 10, along roadside 9 and from bus terminals 11 were collected and analyzed. Most of juice was made from mango (76.7 %) and mixture of fruits (passion, avocado and watermelon). The dominant colour was mango colour (yellow) and all the fruit juice had good smell and there were no dirty materials as contaminants. All the juice samples were acidic with pH range of 3.4–5.1 and 80 % of the vendors handled fruit juice in a clean environment. The container cleanliness assessment revealed that 43.3 % were dirty (Table 3).

**Table 3:** Physicochemical parameters raw fruit juice samples (n = 30)

Variable	Category	Frequency	Percentage
Juice type	Mango	23	76.7
	Mixture	7	23.3
Colour	Yellow	30	100.0
	Others	0	0.0
Odour	Good	30	100.0
	Bad	0	0.0
Presence of dirty in juice	Yes	0	0.0
	No	30	100.0
Cleanliness of juice containers	Dirty	13	43.3
	Satisfactory	7	23.3
	Clean	10	33.3
Cleanliness of the environment at juice selling points	Dirty	6	20.0
	Clean	24	80.0
Juice pH	3–3.9	4	13.0
	4–4.9	24	80.0
	5–5.9	2	7.0

Note that the percentages of juice physicochemical parameters were calculated out of 30 samples analysed

Results for assessment of bacterial load in fruit juice by MPN and TVC are shown in Table 4. The MPN ranged between 460 and ≥1100 at 37 °C, while it ranged between 11 and ≥1100 at 44.5 °C. *E. coli* was identified in 63.3 % of the juice samples examined. Juice samples from bus terminal, roadside and cafeteria were contaminated with *E. coli* at the rates of 26.7, 23.3 and 13.3 %, respectively. The TVC varied from 4.2 to 6.3 Log cfu/ml at 37 °C and 4 to 5.9 Log cfu/ml at 44.5 °C. According to Gulf standards the TVC of any fruit juices should not exceed 3.4–4.7 log cfu/ml at 37 °C [46]. Referring to this standard, 73.3 % of the fruit juice samples had TVC beyond the recommendations (Table 4).

**Table 4:** Fruit juice microbiological results by MPN and TVC results (Log cfu/ml)

Incubation temperature	Total coliform count	Number of fruit juice samples	Percentage	TVC (Log cfu/ml)	Number of fruit juice samples	Percentage
At 37 °C	0	7	23.3	0	8	26.7
	460	1	3.3	4.2–5.0	4	13.3
	1100	2	6.7	5.2–5.4	4	13.3
	≥1100	20	66.7	5.7–5.9	3	10.0
				6.1–6.3	6	20.0
				>6.3	5	16.7
At 44.5 °C	0	8	26.7	0	9	30.0
	460	2	6.7	4–5.1	6	20.0
	11–28	4	13.3	5.2–5.7	6	20.0
	1100	1	3.3	5.8–5.9	4	13.3
	≥1100	15	50.0	>6.3	5	16.7

Based on sources of samples, all the 11 samples collected at bus terminal had high coliform growth at 37 and 45.5 °C and also all samples had high TVC (>6.3 Log cfu/ml). Out of 9 fruit juice samples collected along roadside, 7 had coliforms and other microbial growths at 37 and 45.5 °C. Cafeteria samples had the least TCC and TVC at both temperature range of 37 and 45.5 °C. Assessment of different sources of juice samples against mean TVC and TCC showed no significant difference (*p* > 0.05) with the highest mean TVC (6.5 Log cfu/ml) recorded in juice samples from bus terminal.

### Physical and microbiological quality of fish samples

A total of 185 fish samples were collected from different fish selling points in Morogoro of which, 79 (42.7 %) were marine and 106 (57.3 %) freshwater fish. Fourteen fish shops and Mindu dam shore fish selling points were involved in the study. The marine fish species were *Siganus*, *Sardine*, *Caranx*, *Tuna* and *Lethrinus* while freshwater fish species were tilapia (*Oreochromis niloticus* and *O. urolepsis*) and *Clarias gariepinus*. The physical appearance of the slime layer on fish skin to most fish (62 %) had started becoming turbid, opaque and milky. All the assessed fish had soft muscles with pitting characteristics on touch. The tilapia had their scales easily peeling off. Majority of the fish (80 %) generally appeared dull with loss of bloom, their eyeballs were sunken and cloudy, their gills appeared brown or gray covered with some mucus and others were greenish in colour. Forty-three percent of the fish had sour smell.

The prevalence of water-borne bacteria in fish was 54.6, 0.5 and 0.0 % for *E. coli*, *C. jejuni* and *Salmonella* spp. respectively. The only *C. jejuni* isolated was from freshwater fish (*Tilapia*) sourced from Mtera dam. Freshwater fish and unfrozen fish were more contaminated with *E. coli* than the marine fish and frozen fish respectively (*p* < 0.05) (Table 5). The mean TVC of raw fish was 8.1 (Log cfu/g) with the highest count recorded in freshwater unfrozen fish samples (8.9 Log cfu/g) from Mindu dam (Table 5). The lowest microbial count (4.2 Log cfu/g) was observed in frozen marine fish from Tanga. Comparisons of fish types, storage conditions and sources against differences in the means TVC showed significant differences (*p* = 0.0000). According to Tanzania criteria/guidelines established by Fisheries Act No. 22 of 2003 and Fisheries Regulations of 2003 [47] it recommends that the TVC should not exceed 5.0 Log cfu/g for raw fish. With these results, 96.2 % the fish samples had TVC beyond the recommended limits [47].

**Table 5:** Fish types, storage conditions and sources of raw fish against TVC and *E. coli* contamination (n = 189)

Factor assessed	Category	Mean TVC (Log cfu/g)	p value	Number of fish contaminated by *E. coli*	Percentage	p value
Type of fish	Freshwater	8.3	0.0000	66	35.7	0.0227
	Marine water	6.2		35	18.9	
Storage condition	Frozen	7.2	0.0000	47	25.4	0.0006
	Unfrozen	8.4		54	29.2	
Source of fish	Mindu	8.5		45	24.3	
	Tanga	6.2	0.0000	30	16.2	0.0529
	Mwanza	7.7		12	6.5	
	Mtera	7.7		9	4.9	
	DSM	6.2		5	2.7	

## Discussion

The current study aimed to assess the physical quality and establish the microbial status in raw milk, raw fish and raw unpasteurized fruit juices locally sold in Morogoro Municipality, Tanzania. It was generally found that the milk sold in Morogoro Municipality was of poor quality as depicted through physical and bacteriological assessment. Unpasteurized raw fruit juice indicated high bacterial load which may be sources of spoilage and endangers the health of consumers. Similarly, fish bacterial analysis indicated high prevalence of *E. coli* as an indicator of faecal pollution in water and also isolated *C. jejuni* from freshwater fish. High proportion of food samples with poor physical quality and high bacterial loads as was observed during this study suggests that there is high risk of public exposure to food-borne diseases in Morogoro Municipality. Food producers and vendors should be educated on good and safe food preparations accompanied with good hygienic practices so as to safeguard the public from eating contaminated food. It is further stressed that there is a need for enforcement of regulations regarding the safety of locally vended food and routine inspection of food, selling premises and regular health check-up of the personnel involved in food vending industry.

### Assessment of milk quality

Presence of physical dirt in raw milk serves as potential sources of microbial contamination and cause poor eye appeal to consumers that may lead to lowered marketability. Abnormally bad smell of milk observed during this study partly may be contributed by spoiled milk, dirty containers, contamination with dirty like cow dung, hairs and general poor milk hygiene. Similar observations have been reported by Bukuku et al. [48]. Contaminations accelerate spoilage which may lead to milk-borne diseases to consumers. Large amount (70 %) of the milk sold had low specific gravity (SG) below standard of 1.028 g/ml as given by East African community [34] which suggests adulterations.

The results of the present study showed that (60 %) of all milk handled had higher TVC than the maximum recommended of 5.3 Log cfu/ml as given by East Africa Community standards [34]. The mean TVC of raw milk was 3.4 Log cfu/ml with the highest count recorded in raw unboiled milk samples (5.5–5.6 Log cfu/ml). This suggests a considerable proportion of milk consumers are at risk of milk-borne diseases since majority of the people in Tanzania consume raw unboiled milk [29]. Bacterial contamination of raw milk can occur from three main sources; within the udder, outside the udder, and from the surface coming into contact with the milk [12, 13, 48]. It was observed that among the milk samples with highest TVC were those with sediments (cow dung and hairs) which are potential sources of microbes acquired from the animal itself. In addition, farmers and other milk dealers may incur losses due to rejection of spoiled milk as was observed during this study, 25 % of the milk samples tested clotted on alcohol test. The results of this study are inline to those previously published in Tanzania by Bukuku et al. [48] and Kivaria et al. [49] in which most of the samples tested had higher bacterial count above the standards.

Surprisingly, a high mean TVC of 4.3 Log cfu/ml in boiled milk was recorded implying that boiling is not the only critical step for improving the microbiological quality of milk products. The unsatisfactory quality of boiled milk is the consequence of the poor quality of raw milk used and/or a high level of recontamination after boiling. Poor handling and storage of boiled milk in restaurants/kiosks observed during this study give high possibilities for post-boiling contaminations. These findings highlight the fact that boiled milk of such poor microbiological quality poses a threat to consumers. *E. coli* was isolated from 62.5 % of the milk samples analyzed. According to WHO guidelines, *E. coli* should not be present in any raw or processed milk since the bacteria are used as an indicator of faecal contamination. In addition, strains like *E. coli* O157:H7 are also pathogenic to humans and their occurrence has been reported in Tanzania [50].

### Assessment of fruit juice quality

The general finding on fruit juice showed that it was prepared and served in unhygienic environments. Most of the containers (43.3 %) used to store juice were dirty while 20 % of the selling points were also dirty suggesting of contamination. Disregard of sanitary practices during juice preparation, storage in clean containers and vending in clean environment might be the possible cause of high bacterial load in fruit juices sold along roadsides and bus terminals as previously reported by Nonga et al. and Simforian et al. [8, 11]. This was supported by the high bacterial load in fruit juice samples beyond the recommendations. According to the Gulf standards, the allowable TVC levels for raw unpasteurized fruit juices should be within 3.7–4.0 Log cfu/ml [46]. Majority of the fruit juices (73.3 %) in the current study had TVC above the recommended levels which imply poor quality. The TVC recorded in the current study varied from 4.2 to 6.3 Log cfu/ml at 37 °C and 1–5.8 Log cfu/ml at 44.5 °C which indicate a serious case of poor hygiene and the juice was unfit for consumption. This range is comparable to the findings reported by Simforian et al. [11]; it is however, lower than those reported by Kumar et al. [51]. With these results, it calls for the routine screening and control of locally extracted fresh fruit juice which is being sold in streets by the local vendors in different town centers in Tanzania.

It was further found that majority of the samples were contaminated with coliforms in particular *E. coli* which indicates evidence of faecal contamination or poor hygiene and the juice was unfit for consumption. The main source of coliform contamination might be through contaminated water supplies which are used in processing of juice or the personnel [52]. Assessment of food preparation areas indicated that majority of vendors prepared and sold juices in unhygienic conditions that attracted houseflies and dust. According to Tanzania Specifications (TZS 585:2003), coliforms like *E. coli* must not be present in ready to drink beverages [53]. The *E. coli* prevalence recorded in the current study indicates that most of the raw unpasteurized fruit juice vended in Morogoro is not fit for human consumption and predisposes consumers to food-borne diseases.

### Assessment of raw fish quality

Results on raw fish indicated that most of the fish were of poor quality since most of them were at different stages of spoilage and therefore unfitness for human consumption. High prevalence of *E. coli* (54.6 %) was recorded which indicates evidence of faecal contamination either from the water body, rapture of intestines during eviscerations or contamination along the fish value chain mainly due to poor hygiene and storage conditions. Some strains of *E. coli* are known to cause diarrhea, urinary infections, pyogenic infections and septicemia in humans [50, 54, 55]. Meanwhile, isolation of *C. jejuni* from tilapia sourced from Mtera dam poses more danger to fish consumers. *Campylobacter jejuni* has been reported in Tanzania as among the major causes enteritis in humans [55, 56].

Findings on bacterial enumeration in fish indicated that the mean TVC in freshwater fish was 8.3 Log cfu/g while in marine fish was 6.2 Log cfu/g. According to Tanzania criteria/guidelines established by Fisheries Act No. 22 of 2003 and Fisheries Regulations of 2005 [47] it recommends that the TVC should not exceed 5.0 Log cfu/g meaning that 96.2 % of the fish samples had TVC beyond the recommendations. Other studies in Tanzania have also reported high bacteria count in freshwater and marine fish [20, 57]. High bacterial load in fish predisposes them to fast spoilage and possibly become potential sources of food-borne poisoning to consumers. This was evidenced by the results of physical assessment which showed majority of the fish were spoiled. In addition, presence of pathogenic bacteria like *C. jejuni* in raw fish poses a health risk to the consumers.

## Conclusions

It is concluded that most of food vended in the Morogoro Municipality is of poor quality physically and microbiologically, which poses health risks to public and losses to food owners due to spoilage. Stakeholders in food value chain should be educated on safe production and good hygienic practices. Routine safety assessment of locally vended food, inspection of selling premises and regular health check-up of the personnel involved in food vending industry should be done.

## Abbreviations

BPW: buffered peptone water
COSTECH: Tanzania Commission for Science and technology
FAO: Food and Agriculture Organization
ISO: International Standards Organization
MPN: most probable number
pH: a measure of the acidity or basicity of an aqueous solution
PHCT: Population and Housing Census of Tanzania
SG: specific gravity
SUA: Sokoine University of Agriculture
TBS: Tanzania Bureau of Standards
TCC: total coliform count
TVC: total viable count
TZS: Tanzania specifications
UNIDO: United Nations Industrial Development Organization
WHO: World Health Organization
